# FAMM Flap in Reconstructing Postsurgical Nasopharyngeal Airway Stenosis

**DOI:** 10.1155/2014/276058

**Published:** 2014-09-21

**Authors:** Ferdinand Wanjala Nangole, Stanley Ominde Khainga

**Affiliations:** Department of Surgery (Plastic), University of Nairobi, Nairobi 00200, Kenya

## Abstract

*Introduction*. Postsurgical nasopharyngeal airway stenosis can be a challenge to manage. The stenosis could be as a result of any surgical procedure in the nasopharyngeal region that heals extensive scarring and fibrosis.* Objective*. To evaluate patients with nasopharyngeal stenosis managed with FAMM flap.* Study Design*. Prospective study of patients with nasopharyngeal stenosis at the Kenyatta National Hospital between 2010 and 2013 managed with FAMM flap. *Materials and Methods*. Patients with severe nasopharyngeal airway stenosis were reviewed and managed with FAMM flaps at the Kenyatta National Hospital. Postoperatively they were assessed for symptomatic improvement in respiratory distress, patency of the nasopharyngeal airway, and donor site morbidity.* Results*. A total of 8 patients were managed by the authors in a duration of 4 years with nasopharyngeal stenosis. Five patients were managed with unilateral FAMM flaps in a two-staged surgical procedure. Four patients had complete relieve of the airway obstruction with a patent airway created. One patient had a patent airway created though with only mild improvement in airway obstruction.* Conclusion*. FAMM flap provides an alternative in the management of postsurgical severe nasopharyngeal stenosis. It is a reliable flap that is easy to raise and could provide adequate epithelium for the stenosed pharynx.

## 1. Introduction

Postsurgical nasopharyngeal airway obstruction is a relatively uncommon condition. The condition however when present can result in severe airway obstruction leading to both physical and psychosocial stress to the patients and the guardians. Patients with this condition are forced to breathe through the mouth and thus experience severe sleep apnea. Delayed management of this condition could result in failure to thrive, poor performance in school, and right-sided heart failure.

Management of this condition has traditionally involved relieving the obstruction by surgical or laser excision of scarred tissue [1–4]. The epithelial lining is then provided by either skin grafts, local flaps, or free flaps such as radial forearm flap [5, 6]. In some cases excision of scarred tissues followed by insertion of a stent for a duration of two to three weeks has proven sufficient [7]. Adequate excision of scarred tissue or inadequate epithelial lining after excision of scar tissue would almost always result in the recurrence of the stenosis.

In this presentation we share our experience with the use of the facial artery muscular mucosa flap in the management of the nasopharyngeal stenosis (FAMM) at the Kenyatta National Hospital, a tertiary facility in Kenya.

## 2. Pertinent Literature

Nasopharyngeal stenosis is a relatively rare condition with an incident of about 3 in 100,000 cases after tonsillectomy surgery [8]. Infective causes such as syphilis and other granulomatous disease were a contributory to many cases before the Second World War. With the advent of better antibiotics, infection is not a big contributor to this condition.

Most cases of nasopharyngeal stenosis are due to either surgical procedures in the nasopharyngeal region or postradiation for nasopharyngeal tumours [9, 10]. The common surgical procedures attributed to the causation of nasopharyngeal stenosis are adenoidectomy and uvulopalatoplasties [9, 10]. Laser therapy for adenoids in the nasopharyngeal region has also been attributed to the causation of this disease [11].

Diagnosis of nasopharyngeal stenosis relies mainly on history and physical examination. History of surgery in the nasopharyngeal airway either for adenotonsillectomy, uvulopalatoplasty, and uvulectomy followed by difficulties in nasal breathing is highly suggestive. Other symptoms may be snoring, anosmia, rhinorrhea, otalgia, and dysphagia.

Physical examination will reveal absence or reduction of the nasopharyngeal orifice with scarring of the soft palate and the pharyngeal wall. Nasal endoscopy or postnasal mirror where they are available could assist in confirming the diagnosis.

Management of nasopharyngeal stenosis could be challenging. The condition is prone to high incidence of recurrence in spite of adequate planning and proper techniques.

The main modality in the management of nasopharyngeal stenosis is surgery. The principles entail the following:

1. removal or excision of the scar tissues: this could either be through surgical excision or through the use of carbon dioxide laser,
2. maintaining patency of the orifice until epithelialisation of the orifice tract (by use of stents or obturators),
3. provision of epithelial lining: this could be provided by either skin grafts, local flaps, or regional or free flaps.

Due to the high recurrence rates noted in the management of this condition, many treatment modalities are now available in attempting to prevent the recurrences. The surgical options reported in literature include the use of either soft palate or pharyngeal wall flaps [12–14]. With this the scarred tissue is excised and the mucosal flaps either from the soft palate or the posterior pharyngeal wall mobilized to cover the defect. Other flaps reported in literature include the sternocleidomastoid myocutaneous flap [15]. Skin grafts and free flaps have also been used in the management of this condition with varying degrees of success [6].

Carbon dioxide laser has increasingly been utilized in the management of this condition. Krespi and Kacker probably report the largest series in the use of laser in the management of nasopharyngeal stenosis with good outcome [5]. Postexcision of an obturator is utilized by the patient for 2 to 6 months to prevent recurrence. Igwe et al. have utilized carbon dioxide laser to make radial cuts in the stenosed nasopharynx and then utilize controlled balloon dilatation to continuously expand the orifice [16]. Mitomycin c is then topically applied over the scar area to prevent fibroblast activities.

Plasma hook with mitomycin c as a modality for treatment of nasopharyngeal stenosis has also been reported with Madgy et al., though being only in a series of three patients [17].

Other nonsurgical techniques in the management of nasopharyngeal stenosis include the use of radiofrequency waves with or without mitomycin c. Hussein et al. with the use of radiofrequency and mitomycin c still advocated for the use of stents two weeks after surgery [18]. Wang et al. on the other hand found that this technique was best suited for patients with at least three months after the development of the stenosis [19].

## 3. Materials and Method

Patients with nasopharyngeal stenosis managed in the multidisciplinary clinic at the Kenyatta National Hospital were followed up during the study duration of four years between Jan 2010 and December 2013. Information gathered included the patients' demography, etiological cause of the nasopharyngeal airway obstructions, and any previous surgical interventions. A detailed history and clinical evaluation were then done to determine the severity of the airway obstructions. The severity of the obstruction was graded as follows.

*Grade 1.* Mild airway obstruction: these were patients who reported a good night sleep with only occasional episodes of dyspnoea. They predominantly breathe through the nose.

*Grade 2.* Moderately airway obstruction: these were patients who predominantly breathe through the nose during the day but had several episodes of nocturnal dyspnoea that disrupted their sleep pattern.

*Grade 3.* Severe airway obstruction: these were patients who could only breathe through the mouth. They persistently had sleep apnea and had other manifestations such as failure to thrive, poor school performance, and difficulties in feeding. Patients with previous surgical who attempt to relieve the stenosis with no success were also considered in this grade.

Patients who were classified in grade 3 were managed by the FAMM flap. The other patients in grades 1 and 2 were managed by the local flaps.

**Figure 1:**
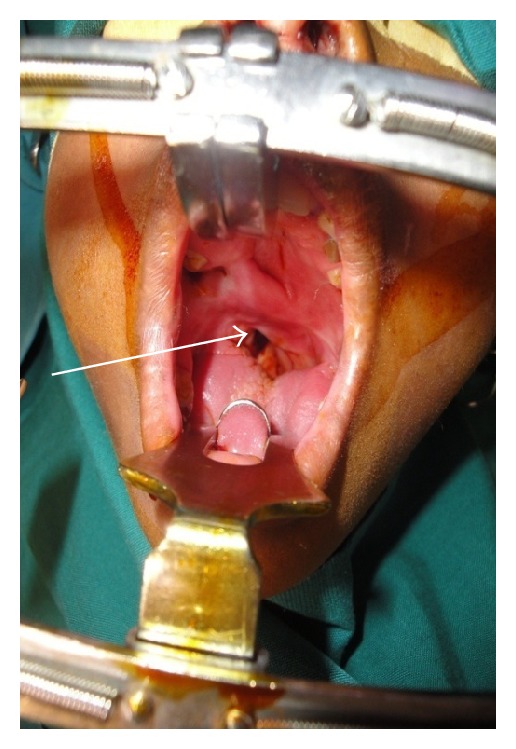
Patients with severe nasopharyngeal stenosis with extensive scarring of the soft palate.

**Figure 2:**
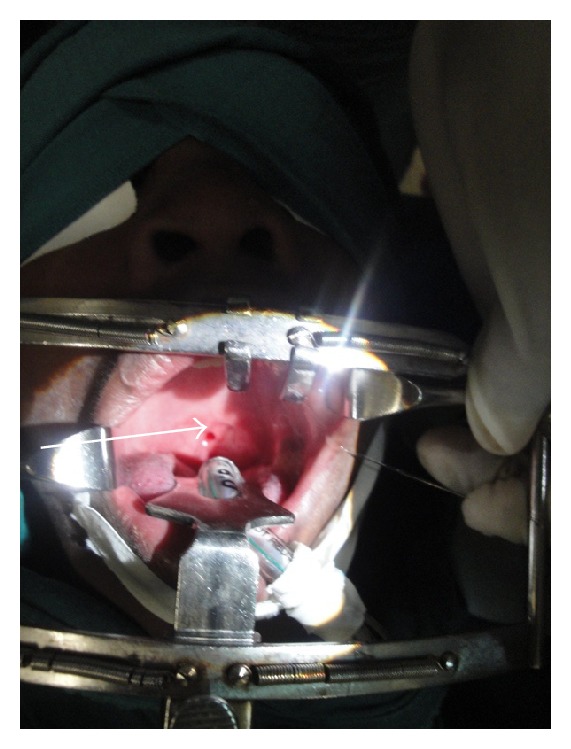
Patients with severe nasopharyngeal stenosis with extensive scarring of the soft palate.

**Figure 3:**
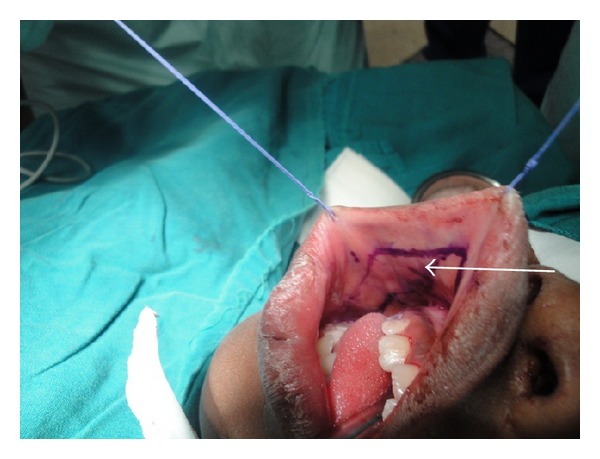
Markings for the FAMM FLAP.

Operatively all the patients had oral intubation. Nasoendoscopy was done to confirm the presence of the stenosis. Tongue retractor was utilized to retract the tongue and keep the mouth wide open. Throat pack was inserted into the oral pharynx. After infiltration of lignocaine with adrenaline solution, the scarred tissue was excised until complete patency of the nasopharyngeal orifice was achieved. The facial artery was identified in the oral mucosa with the aid of hand held Doppler. Inferiorly based pedicle FAMM flap was then raised with the width of the flap corresponding to the extent of the defect to be covered (Figures 1 and 2). The dissection was carried out in the plane deep to the buccinators muscle. The parotid duct was identified and necessary precautions were taken not to injure it. The dissected flap was then advanced into the raw area over the soft palate ensuring complete separation of the soft palate from the nasopharyngeal wall (Figure 3). Suturing of the flap commenced between the flap and the soft palate nasal mucosa with interrupted Vicryl 2/0. This was followed by suturing the flap with soft palate oral mucosa. Upon completion nasogastric tube was passed through either of nostrils to confirm patency of the nasal airway. The donor site was closed primarily. Postoperatively the patient was intubated overnight in the intensive care unit to ensure patency of the airway.

**Figure 4:**
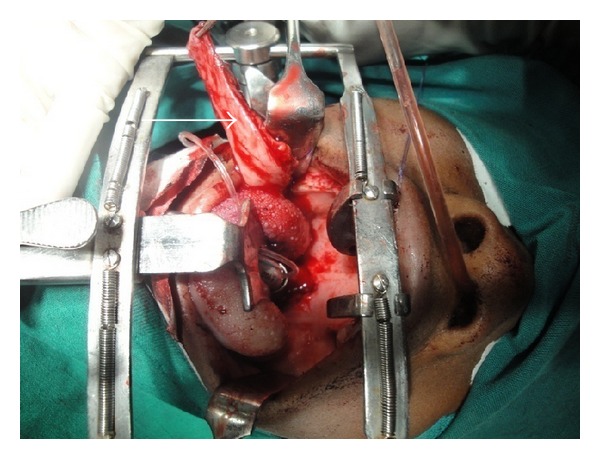
FAMM flap is raised.

**Figure 5:**
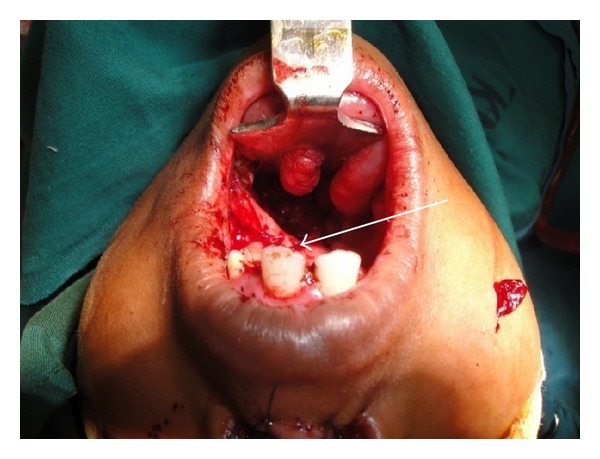
Flap inserted into the defect over the soft palate and pharyngeal wall.

The second stage procedure was done at about three weeks after the first surgery (Figure 4). Upon intubation patency of the nasal airway was checked by passing a nasogastric tube through either of the nostrils. The flap was detached from the buccal region and the facial artery ligated. Any redundant flap tissues were excised and proper positioning of the flap done (Figure 5).

After surgery, clinical evaluation of the patients was done by assessing improvement in airway obstruction, patency of the nasal airway, and healing of the donor site. Improvement in airway obstruction was graded as

1. mild improvement: only slight improvement in airway obstruction with the patient still having predominantly oral breathing and sleep apnoea,
2. moderate improvement: patient predominantly breathes through the nose but still has some episodes of sleep apnoea at night,
3. great improvement: patient does not experience any more sleep apnoea and breathes through the nose.

The patients were followed up for at least one year in the clinic to check for any evidence of recurrence.

## 4. Results 

A total of 8 patients with nasopharyngeal stenosis were reviewed in the multidisciplinary clinic at the Kenyatta National Hospital during the study duration of four years. Six patients had developed stenosis after traditional uvulectomy with 2 patients after adenoidectomy. The age range for the patients was 4 years to 12 years of age. Five were female patients with 3 male patients. Of the 8 patients seen 5 patients were classified as grade 3, with severe nasopharyngeal stenosis. The other 3 patients were classified as follows: grade 1, one patient, and grade 2, 2 patients. Patients with grades 1 and 2 (*n* = 3) were managed by excision of scar tissues and local pharyngeal flaps. All grade 3 patients (*n* = 5) had had an average of two surgical procedures elsewhere before referral to our clinic. They were all managed with unilateral inferiorly based FAMM flap in two staged procedures. Of the five patients managed with the FAMM flaps, four patients had complete relieve of the nasal airway obstruction with no sleep apnoea at one year of follow-up. One patient had mild improvement in the airway obstruction. All patients had patent nasal airway at one year of follow-up. The donor sites in all patients were closed primarily and healed with no complications. No facial nerve palsy nor injury to the parotid duct was noted in any patient. There was no flap loss or necrosis in any of the patients either.

## 5. Discussion 

Traditional uvulectomy contributes to a big proportion of patients with nasopharyngeal stenosis in our setup. The belief in many African communities is that the uvula is responsible for recurrent respiratory tract infections especially in the pediatric age groups. The uvula should thus be excised to save the child from persistent throat or airway infections. Uvulectomy unfortunately in many communities is done in unhygienic conditions resulting in infections with resultant scarring of the nasopharynx with severe stenosis as seen in the majority of the patients in this study. There is thus a strong need for community education on the possible complications of this practice.

Nasopharyngeal stenosis in the literature could be managed by carbon dioxide laser for excision of scarred tissues [2–4]. After excision the patency of the airway is maintained by either a stent or an obturator for up to 6 months while waiting for epithelialisation of the orifice. Laser despite the fact that it is now widely used in the medical field is however not readily available in many countries like ours where resources are constrained. Our unit thus has no experience whatsoever on the management of nasopharyngeal stenosis with this modalities. It should however be noted that even in centers where it is widely practiced the patient still has to use a stent or an obturator for up to 6 months to prevent any recurrence. Mitomycin c may also have to be applied on the scar tissue after laser so as to suppress fibroblast activities. FAMM flap on the other hand provides adequate epithelial lining that allows tissues to heal by primary intention and hence there is no risk of recurrence due to healing by secondary intention. There is thus no need to use a stent or an obturator after surgical excision.

Surgical management of nasopharyngeal stenosis basically entails excision of the scar tissue with provision of an epithelial lining. A stent or an obturator may be utilized depending on the practice in the center. After excision of the scarred tissues, the epithelial ling could be provided by either skin grafts, local flaps, regional flaps, or even free flaps [12, 13]. The choice of which option to use could be influenced by many factors including severity of stenosis, quality of the surrounding mucosa, operating surgeons experience, and possible complications associated with the method chosen.

Skin graft is probably an easy option in the provision of epithelial lining anywhere on the body. It is easy to harvest with minimal donor site morbidity. Its biggest disadvantage however in the management of patient with nasopharyngeal stenosis is that the graft tends to contract and hence is prone to recurrence of the stenosis. It is also technically difficult to successfully secure the graft at the recipient site with pressure dressings and thus one is likely to experience poor graft take. Local pharyngeal and soft palatal flaps are probably the most quoted flaps in literature utilized in the management of nasopharyngeal stenosis [12–14]. These flaps have an advantage that they are proximal to the defect being reconstructed and thus are easier and faster to rise with minimal donor site morbidity.

Our experience however is such that they are probably only useful in patients with mild or moderate nasopharyngeal stenosis. Patients with severe stenosis usually have extensive scarring involving the mucosa and underlying tissues. It is thus technically difficult to raise such flaps and reconstruct defects appropriately. The flaps are also limited as to how much epithelial surface they could provide.

Regional flaps quoted in literature in the management of nasopharyngeal stenosis include the sternocleidomastoid musculocutaneous flap [15]. A possible shortcoming with this flap is an external skin incision that may not be cosmetically acceptable especially to our patients who have a tendency to form hypertrophic scars and keloids. Being a muscle flap it is also likely to be bulky and thus obliterate the nasopharyngeal orifice.

Facial artery musculomucosal flap (FAMM) is a pedicle flap that could be proximally or distally raised based on the facial artery. The facial artery is identified by a hand held Doppler and it is location mapped in the flap. The flap is able to provide mucosa lining of up to 3 cm in dimension while allowing for the closure of the donor site primarily [20]. This flap is well established in the reconstruction of multiple defects including palatal, nostril, nasal septal, and upper lip [20, 21].

Due to its wide arc of rotation the flap once fully mobilized is able to reach most parts of the soft palate and the nasopharynx. It is thus able to cover most of the raw area in patients with severe nasopharyngeal stenosis once the scar tissues are excised, providing adequate epithelial lining and hence preventing recurrence of the stenosis. The flap is also thin and pliable, unlike most muscle or free flaps. It only provides mucosa with minimal muscle bulk to the scarred area. Due to its rich blood supply, flap necrosis is uncommon. The donor site in the majority of the patients heals without any complications.

**Figure 6:**
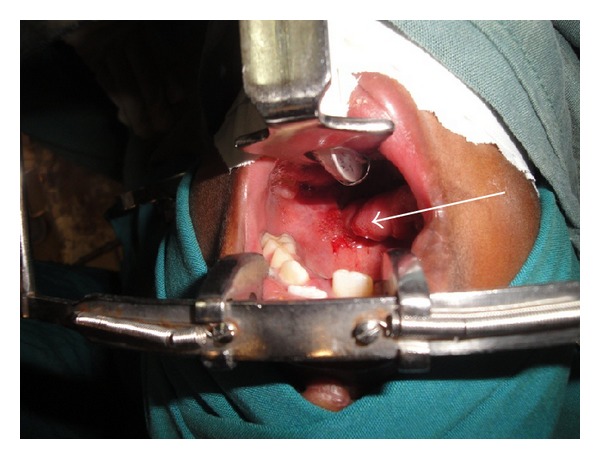
Patient with FAMM flap for the second staged procedure arrow indicating the redundant flap tissue around the pedicle to be excised

**Figure 7:**
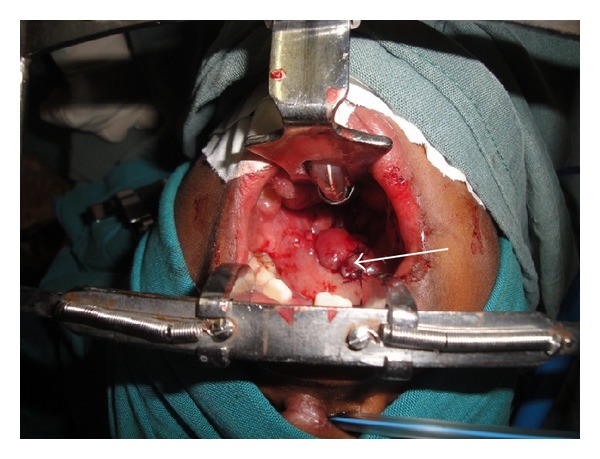
Patient after detaching the flap and excision of redundant tissues. Note patent airway as demonstrated by the nasogastric tube.

In our patients a two-staged procedure was done. The first stage encompassed raising the flap and advancing it into the defect with the second stage detaching the flap from its donor site and excising any redundant tissues. This allows for proper insertion of the flap and removal of any unnecessary tissues in the oral cavity (see Figures 6 and 7). Neither stents nor obturators were utilized since the epithelial surface was already established with the flap.

All our patients at one year of follow-up had a patent nasal passage with no recurrence of stenosis. However one patient had only mild improvement in his airway obstruction symptoms. This patient was however a syndromic patient with midface hypoplasia.

In conclusion FAMM flap provides an alternative in the management of nasopharyngeal stenosis. It is a relatively easy flap to raise and has less donor site morbidity. The flap is able to provide adequate epithelial lining of the scarred raw soft palatal region and thus reduce chances of healing by scarring. It should thus be considered in the management of patient with severe nasopharyngeal stenosis with extensive scarring of the pharynx and the soft palate.
